# Prostaglandin E2 receptor type 2-selective agonist prevents the degeneration of articular cartilage in rabbit knees with traumatic instability

**DOI:** 10.1186/ar3460

**Published:** 2011-09-14

**Authors:** Hiroto Mitsui, Tomoki Aoyama, Moritoshi Furu, Kinya Ito, Yonghui Jin, Takayuki Maruyama, Toshiya Kanaji, Shinsei Fujimura, Hikaru Sugihara, Akio Nishiura, Takanobu Otsuka, Takashi Nakamura, Junya Toguchida

**Affiliations:** 1 Department of Tissue Regeneration, Institute for Frontier Medical Sciences, Kyoto University, 53 Kawahara-cho, Shogoin, Sakyo-ku, Kyoto 606-8507, Japan; 2 Department of Orthopaedic Surgery, Graduate School of Medicine, Kyoto University, 54 Kawahara-cho, Shogoin, Sakyo-ku, Kyoto 606-8507, Japan; 3 Department of Orthopaedic Surgery, Graduate School of Medical Sciences, Nagoya City University, Nagoya, Japan; 4 Department of Physical Therapy, Human Health Sciences, Graduate School of Medicine, Kyoto University, 53 Kawahara-cho, Shogoin, Sakyo-ku, Kyoto 606-8507, Japan; 5 Ono Pharmaceutical Co. Ltd.,3-1-1 Sakurai, Shimamoto-cho, Mishima-gun, Osaka 618-8585, Japan; 6 Center for iPS Cell Research and Application, Kyoto University, 53 Kawahara-cho, Shogoin, Sakyo-ku, Kyoto 606-8507, Japan

**Keywords:** prostaglandin E2, EP_2_, ONO-8815Ly, osteoarthritis, ACLMT

## Abstract

**Introduction:**

Osteoarthritis (OA) is a common cause of disability in older adults. We have previously reported that an agonist for subtypes EP2 of the prostaglandin E2 receptor (an EP2 agonist) promotes the regeneration of chondral and osteochondral defects. The purpose of the current study is to analyze the effect of this agonist on articular cartilage in a model of traumatic degeneration.

**Methods:**

The model of traumatic degeneration was established through transection of the anterior cruciate ligament and partial resection of the medial meniscus of the rabbits. Rabbits were divided into 5 groups; G-S (sham operation), G-C (no further treatment), G-0, G-80, and G-400 (single intra-articular administration of gelatin hydrogel containing 0, 80, and 400 μg of the specific EP2 agonist, ONO-8815Ly, respectively). Degeneration of the articular cartilage was evaluated at 2 or 12 weeks after the operation.

**Results:**

ONO-8815Ly prevented cartilage degeneration at 2 weeks, which was associated with the inhibition of matrix metalloproteinase-13 (MMP-13) expression. The effect of ONO-8815Ly failed to last, and no effects were observed at 12 weeks after the operation.

**Conclusions:**

Stimulation of prostaglandin E2 (PGE2) via EP2 prevents degeneration of the articular cartilage during the early stages. With a system to deliver it long term, the EP2 agonist could be a new therapeutic tool for OA.

## Introduction

Osteoarthritis (OA) is the single most common cause of disability in older adults [1]. It is a complex process involving a combination of cartilage degradation, repair, and inflammation. However, its pathogenesis is not yet fully understood [2]. Articular cartilage is composed of chondrocytes, and an extensive extracellular matrix (ECM). The major ECM components are type II collagen and aggrecan. In normal cartilage, catabolic and anabolic activities are in dynamic equilibrium. Chondrocytes can produce several catabolic cytokines such as IL-1 and TNF-α, which in turn induce the production of proteinases including matrix metalloproteinases (MMPs) and disintegrin-like and metalloproteinase with thrombospondin, that lead to the destruction of the matrix network [3,4]. Among the MMPs, MMP-13 (collagenase 3) plays a particularly important role in causing OA [5]. Indeed, transgenic mice carrying an inducible human *MMP-13 *gene develop pathological changes similar to those observed in human OA patients, when the transgene is expressed in articular cartilages of postnatal mice [6]. Moreover, inhibitors of MMP-13 prevent the degradation of articular cartilage [5,7]. Chondrocytes also produce anabolic cytokines such as the bone morphogenetic protein family members and insulin-like growth factor-1 (IGF-1), which induce the synthesis of collagen and initiate the proliferation of chondrocytes [3]. A disruption of the equilibrium between the catabolic and anabolic activities results in catastrophic damage to the articular cartilage, ultimately inducing the pathological condition known as OA.

Prostanoids, including prostaglandin (PG) D2, PGE1, PGE2, PGF2α, prostacyclin (PGI2), and thromboxane A2, are lipid mediators produced in a sequence of cyclooxygenase (COX) -1, -2-catalyzed reactions [8]. The role of PGE2 in the development of OA is controversial. Some reports point to an important role in inflammation [9]. Pro-inflammatory signaling mediators such as IL-1 and TNF-α induce the synthesis of PGE2 by promoting the expression or activities of COX-2 and microsomal PGE synthase-1 [10]. PGE2 then promotes IL-1 expression as part of a positive feedback mechanism, degrades the cartilage ECM [4,10-13], and finally induces apoptosis of chondrocytes [3]. Other reports insist that PGE2 opposes the effect of IL-1 [14] and stimulates the gene expression of type II collagen [3,15]. In addition, PGE2 stimulates the synthesis of proteoglycan and collagen through the expression of an IGF-1-binding protein [16,17]. PGE2 works through four isoforms of the EP receptor, EP1 to EP4. Previously, we considered that the controversy could result from differences in the mode of action and tissue distribution of each receptor [18]. Using an EP2 selective agonist, we showed that EP2 receptor-mediated PGE2 signaling enhances the growth of chondrocytes [18,19] and promotes the regeneration of articular cartilage in rabbits with cartilage defects [19].

In the current study, we investigate the effect of an EP2 agonist on articular cartilage in a rabbit model of traumatic degeneration.

## Materials and methods

### Materials

**Figure 1:**
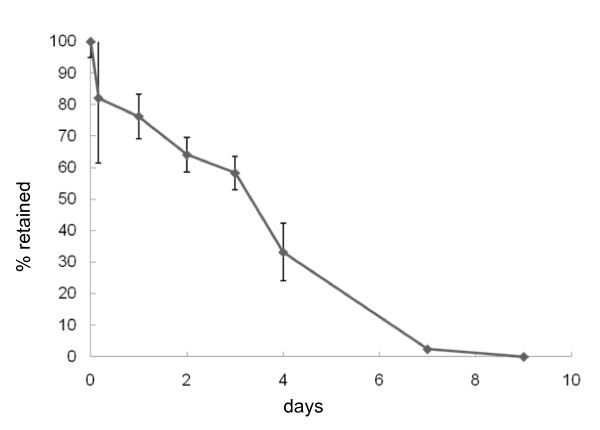
**The diffusion kinetics of ONO-8815Ly from microspheres *in vitro***. Microspheres loading ONO-8815Ly were soaked in PBS, and the amount of retained ONO-8815Ly at each time point was measured by high-performance liquid chromatography and calculated as the ratio to the initial amount (n = 5).

Microspheres loaded with a selective EP2 agonist, ONO-8815Ly (lysine salt) [20], were prepared by the emulsion-solvent evaporation method [19,21]. Briefly, ONO-8815Ly and polylactic-co-glycolic acid (PLGA) were mixed to form a water/oil emulsion, and added to the outer water phase containing polyvinyl alcohol under stirring with a turbine-shaped mixer at 5000 rpm to obtain a water/oil/water emulsion. PLGA microspheres that did not contain ONO-8815Ly in its free form were recovered by centrifugation and lyophilized to remove residual organic solvent and water. Then, a gelatin aqueous solution (20%, w/w) was poured into the microsphere suspension to form a gel. For the crosslink reaction, a glutaraldehyde aqueous solution (12.5 mg/ml) was poured into the microsphere suspension. Small cylinder-shaped gelatin hydrogels (4 mm in diameter and 2 mm in thickness) containing ONO-8815Ly (0, 80, or 400 μg of ONO-8815/gel) were obtained by hollowing out the gelatin hydrogel sheet. Diffusion kinetics analyses showed that ONO-8815Ly is gradually released from the microsphere over a period of seven days *in vitro *(Figure 1).

### Animal model for traumatic degeneration

Four-month-old female Japanese white rabbits (weighing approximately 3 kg) were used. Traumatic degeneration was induced as described for the anterior cruciate ligament and menisectomy transection (ACLMT) model [22]. Operations were performed under general anesthesia, and a skin incision was made on the medial side of the patella. Soft tissues and articular capsules were cut to expose the knee joints. The anterior cruciate ligament was transected at the attachment to the tibia in the knee-flexed position, and the anterior horn of the medial meniscus was resected. The articular capsule and skin were sutured in layers with 4-0 nylon sutures. After the operation, rabbits were allowed to move freely. Preliminary experiments revealed that osteoarthritic changes were observed in this model at as early as two weeks after operation (data not shown).

### Treatments with the EP2-agonist

A total of 64 animals were randomly assigned to five groups: G-S (sham operation), G-C (no further treatment), G-0, G-80, and G-400 (single intra-articular administration of gelatin hydrogel containing 0, 80, and 400 μg of ONO-8815Ly, respectively). Sham-operated rabbits (G-S; n = 4) received no further treatment, and were sacrificed either 2 (n = 2) or 12 weeks (n = 2) after the operation.

The ACLMT surgery was performed on both the knees of each of the remaining 60 rabbits to avoid any unequal bearing of weight due to pain on one side. No further treatment was performed in animals of the control group (G-C; n = 12). In the treatment groups, no further treatment was performed on the right knee, but a gelatin hydrogel cylinder containing ONO-8815Ly (G-0, G-80, and G-400; n = 16 per group) was placed on the fatty pad of the left knee at the time of operation. Rabbits were sacrificed two weeks (G-C, n = 6; G-0, G-80, and G-400, n = 10 per group) or 12 weeks (n = 6 per group) after the operation. All the experiments with animals were approved by the institutional animal research committee, and performed according to the Guidelines for Animal Experiments of Kyoto University.

### Histological examination

Rabbits were sacrificed 2 or 12 weeks after surgery, and the distal femur and proximal tibia of the left side of each animal were resected, fixed at 4°C overnight in a 10% formalin solution, and decalcified in formic acid for three days. After neutralization by 10% sodium sulfate for 24 hours, the samples were embedded in paraffin. Serial sections were prepared in the coronal plane through the middle of the femoral and tibia condyles, and one section from each sample was used for each of the histological analyses. In every section, the entire cartilage portion in full depth was evaluated. The specimens were stained with safranin O/Fast Green or H&E using standard procedures. The histological grade of cartilage degeneration was evaluated using the modified Mankin's scoring system [23], which was adopted as the original system [24] for the evaluation of the rabbit model. All the results shown herein represent the combined scoring data of two researchers.

### Immunohistochemical analyses

Immunohistochemical examination was performed as follows. In brief, after deparaffinization, sections were incubated with 0.3% hydrogen peroxide for 30 minutes. Then, sections were treated with proteinase K for two minutes (proliferating cell nuclear antigen [PCNA] staining) or with hyaluronidase for 60 minutes (MMP staining), after which they were incubated with the following primary antibodies: mouse anti-human PCNA monoclonal antibody (1:100; Dako, Glostrup, Denmark), mouse anti-human MMP-13 monoclonal antibody (1:20; AnaSpec Inc., San Jose, CA, USA), or mouse anti-rabbit MMP-3 monoclonal antibody (1:50; Daiichi Fine Chemical Co. Toyama, Japan). All antibody dilutions were made in PBS. After an overnight reaction with the primary antibody at 4°C, sections were incubated with horseradish peroxidase-conjugated anti- mouse IgG (Vector Laboratories, Southfield, MI, USA) at room temperature for 30 minutes. Signals were visualized with 3, 3'-diaminobenzidine tetrahydrochloride, and nuclei were counterstained with hematoxylin. The percentage of PCNA-, MMP-13-, and MMP-3-positive cells in the cartilage was calculated by methods similar to those described above. Results of histological and immunohistochemical analyses were evaluated by two observers who were blinded to the identity of each sample.

### Primary chondrocyte cultures

Primary culture of chondrocytes was performed using articular cartilage tissues harvested from non-treated rabbits (NRC cells) or ACLMT-operated rabbits (ORC cells). Briefly, thinly sliced cartilage tissues were incubated with collagenase (4 mg/ml; Sigma Aldrich, St. Louis, MO, USA) in DMEM for 12 hours. Cells were then collected by centrifugation, seeded into type I collagen-coated dish (Corning International K.K.,Tokyo, Japan), and cultured with DMEM containing 10% FBS supplemented with 100 units/ml penicillin and 100 mg/ml streptomycin at 37°C in a humidified atmosphere of 5% CO_2_/95% air. Chondrocytes were grown in monolayer cultures, and were passaged when reaching confluence. Cells at the second passage were used for the assay. ONO-AE1-259-01, a selective agonist of EP2, was used to stimulate EP2 signaling in the presence or absence of IL-1β (Sigma Aldrich, St. Louis, MO, USA).

### Real-time PCR

Total RNA was extracted from cultured cells using the RNeasy kit (Qiagen, Valencia, CA, USA) according to the manufacturer's protocol. All reverse transcription reactions were performed with an RT-PCR kit using 1 μg of total RNA with a Superscript II reverse transcriptase (Invitrogen, Carlsbad, CA, USA) for conversion into cDNA. The mRNA expression levels of *MMP-13 *and glyceraldehyde 3-phosphate dehydrogenase (*GAPDH*) were quantified by real-time PCR using SYBR Green (Applied Biosystems, Foster City, CA, USA) and the ABI 7500 Real-Time PCR System (Applied Biosystems, Foster City, CA, USA). All reactions were run in triplicate, and the amount of PCR product of each gene was calculated using the standard curve method and normalized to *GAPDH *levels, which were used as an internal control. Using the ratio obtained for the untreated sample as a standard (1.0), the relative ratio of the treated samples was presented as the relative expression levels of the *MMP-13 *gene. Sequences of primers used in this experiment were as follows: 5'-aggagcatggcgacttctac-3' and 5'-taaaacagctccgcatcaa-3' (*MMP-13*) and 5'-gctctccagaacatcactcctgcc-3' and 5'-cgttgtcataccaggaaatgagct-3' (*GAPDH*).

### Statistical analysis

The statistical analyses were performed using the Statcel2 software (The publisher OMS Ltd., Saitama, Japan). The results are shown as the mean ± standard deviation (SD). The Kruskal-Wallis test was performed for screening purposes, and the Steel-Dwass method for multiple comparisons was used if there was a significant difference between samples. A *P *value less than 0.05 was considered to be significant.

## Results

### Therapeutic effect of ONO-8815Ly in the early stages of degeneration

**Figure 2:**
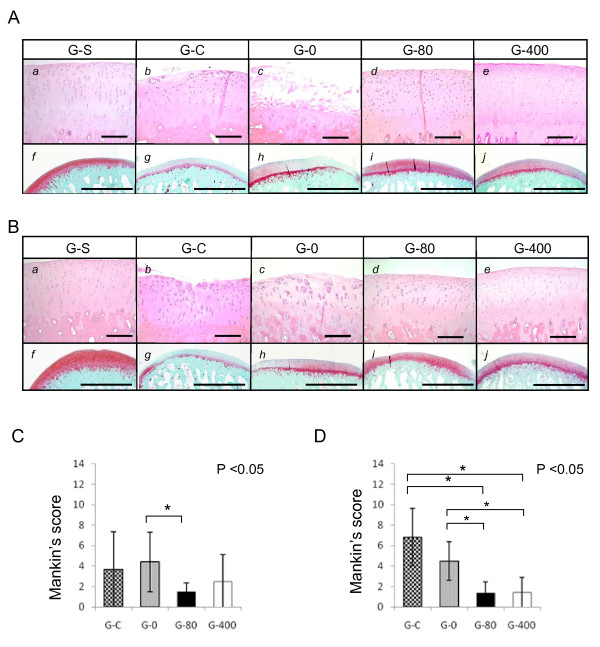
**Effect of ONO-8815Ly in the early stages of OA (femur)**. Histological findings and grade of degeneration, scored using Mankin's system, in (**a**) medial and (**b**) lateral femoral condyles at two weeks after the operation. Sections were prepared from samples of (*a *and *f*) G-S, (*b *and *g*) G-C, (*c *and *h*) G-0, (*d *and *i*) G-80, and (*e *and *j*) G-400, and stained with (*a *to *e*) H&E or (*f *to *j*) safranin-O. Magnification, 200× (H&E) and 40× (safranin-O). Scale bar, 200 μm (HE) and 2.0 mm (safranin-O). *P < 0.05. Values are the mean ± standard deviation. OA, osteoarthritis.

At two weeks after the operation, articular cartilages in medial condyles of G-C (Figure 2a, b) and G-0 (Figure 2a, c) showed severe degenerative findings such as surface irregularity including clefts and reactive changes such as clonal proliferation of chondrocytes. The intensity of safranin O staining was reduced in G-C (Figure 2a, g) and G-0 (Figure 2a, h). The grade of degenerative findings was less prominent in sections of G-S (Figure 2a, a), G-80 (Figure 2a, d) and G-400 (Figure 2a, e) than in those of G-C or G-0. Safranin O staining was stronger in sections of G-80 (Figure 2a, i) and G-400 (Figure 2a, j). Similar findings were observed in sections prepared from lateral femoral condyles. The degenerative changes were less prominent and the safranin O staining was stronger in sections of G-S (Figure 2b a and 2f), G-80 (Figure 2b, d and 2i) and G-400 (Figure 2b, e and 2j) than in those of G-C (Figure 2b, b and 2g) or G-0 (Figure 2b, c and 2h).

Histological grade was evaluated using a modified Mankin's scoring system [23,24]. The grades of medial condyle in each sample were scored and mean values were compared (Figure 2c). Scores were significantly better for G-80 than for G-0. The effect of ONO-8815Ly was more prominent in lateral condyles, and both G-80 and G-400 showed much better scores than G-C or G-0 (Figure 2d).

**Figure 3:**
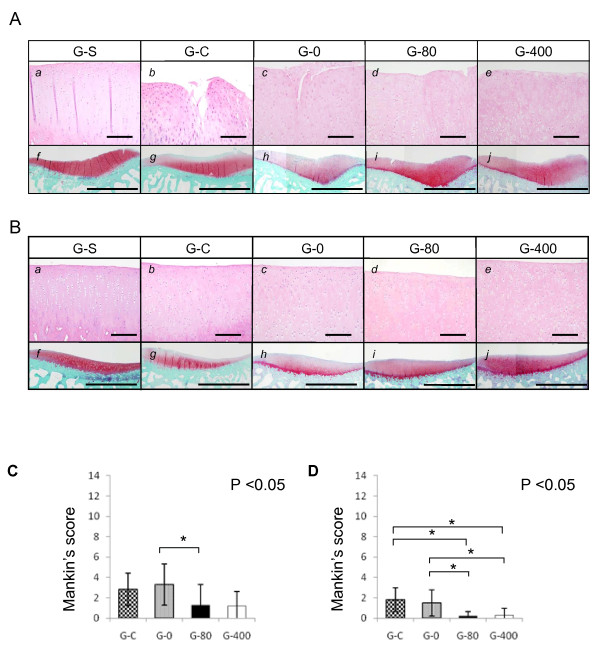
**Effect of ONO-8815Ly in the early stages of OA (tibia)**. Histological findings and grade of degeneration, scored using Mankin's system, in (**a**) medial and (**b**) lateral tibial condyles at two weeks after the operation. Sections were prepared from samples of (*a *and *f*) G-S, (*b *and *g*) G-C, (*c *and *h*) G-0, (*d *and *i*) G-80, and (*e *and *j*) G-400, and stained with (*a *to *e*) H&E or (*f *to *j*) safranin-O. Magnification, 200× (H&E) and 40× (safranin-O). Scale bar, 200 μm (H&E) and 2.0 mm (safranin-O). *P < 0.05. Values are the mean ± standard deviation. OA, osteoarthritis.

Similar findings were observed in medial (Figure 3a) and lateral (Figure 3b) condyles of tibiae. The degenerative changes were less prominent and the safranin O staining was stronger in sections of ONO-8815Ly-treated groups (G-80 and G-400) than in those of non-treated groups (G-C and G-0). The effect of ONO-8815Ly was similar between G-0 and G-80 in medial condyles (Figure 3c), whereas G-80 and G-400 showed better values than G-C or G-0 in lateral condyles (Figure 3d). These results suggested that ONO-8815Ly prevents degenerative change in articular cartilages during the early stages.

### Therapeutic effect of ONO-8815Ly in the late stages of degeneration

**Figure 4:**
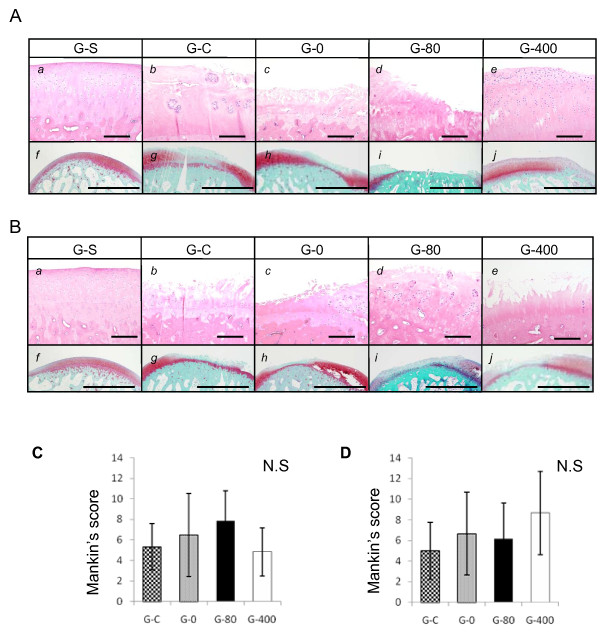
**Effect of ONO-8815Ly in the late stages of degeneration (femur)**. Histological findings of **(a) **medial and **(b) **lateral femoral condyles at 12 weeks after the operation. Sections were prepared from samples of (*a *and *f*) G-S, (*b *and *g*) G-C, (*c *and *h*) G-0, (*d *and *i*) G-80, and (*e *and *j*) G-400, and stained with (*a *to *e*) H&E or (*f *to *j*) safranin-O. Magnification, 200× (H&E) and 40× (safranin-O). Scale bar, 200 μm (H&E) and 2.0 mm (safranin-O). Grade of degeneration scored using Mankin's system in **(c) **medial and **(d) **lateral femoral condyles at 12 weeks after the operation. Values are the mean ± standard deviation. N.S, not significant.

Similar analyses were performed using sections prepared at 12 weeks after surgery. In the case of femoral condyles, no improvements of cartilage degeneration were observed in sections of ONO-8815Ly-treated groups (G-80 or G-400) (Figure 4a, d and 4e) and the staining of safranin O also showed no difference (Figure 4a, i and 4j). Similar results were obtained in lateral condyles of femora (Figure 4b). In agreement, there was no significant difference in Mankin's score in the analyses of medial (Figure 4c) or lateral (Figure 4d) condyles of femora.

Similar results were obtained in the tibiae. Neither medial nor lateral condyles showed better histological features by the treatment with ONO-8815Ly, and the Mankin's score showed no improvements (data not shown).

These results suggested that the effect of ONO-8815Ly failed to last, at least when using this drug delivery system.

### Growth promoting effect of ONO-8815Ly

**Figure 5:**
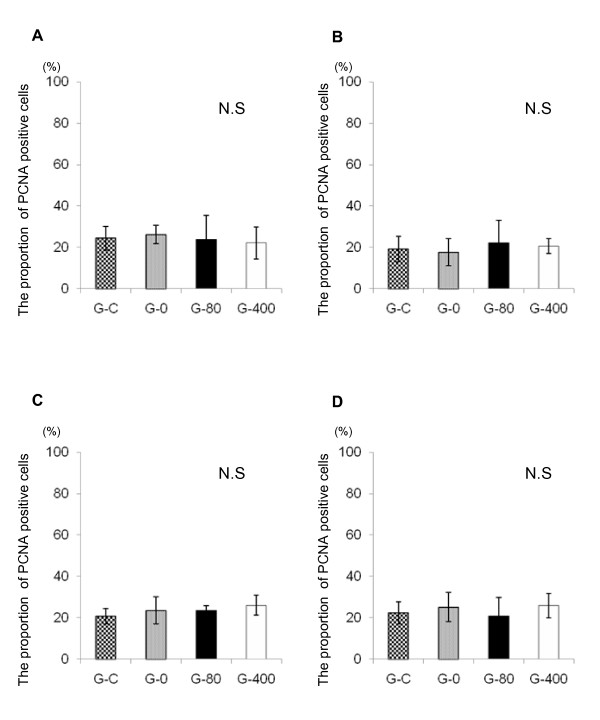
**Effect of ONO-8815Ly on PCNA expression**. The proportion of PCNA-positive cells in **(a) **medial and **(b) **lateral femoral condyles, and in **(c) **medial and **(d) **lateral tibial condyles. Values are the mean ± standard deviation. N.S, not significant; PCNA, proliferating cell nuclear antigen.

The proliferating activity of chondrocytes was evaluated by PCNA staining (Figure 5). The proportion of PCNA-positive cells in femoral (Figures 5a and 5b) and in tibial (Figures 5c and 5d) condyles at two weeks after operation were similar among all groups, suggesting that the improvement of cartilage degeneration by the EP2 agonist was not due to the acceleration of cell proliferation.

### EP2- selective agonist inhibits the expression of MMP-13 in ACLMT

**Figure 6:**
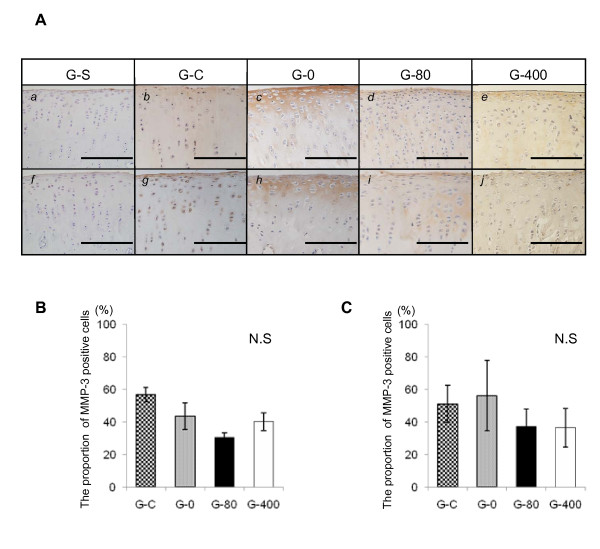
**Effect of ONO-8815Ly on the expression of MMP-3 in femoral condyles**. **(a) **Immunostaining for MMP-3 at two weeks after the operation. Sections of (*a *to *e*) medial and (*f *to *j*) lateral femoral condyles were prepared from samples of (*a *and *f*) G-S, (*b *and *g*) G-C, (*c *and *h*) G-0, (*d *and *i*) G-80, and (*e *and *j*) G-400. Magnification, 400×. Scale bar, 200 μm. Proportion of MMP-3-positive cells in **(b) **medial and **(c) **lateral femoral condyles at two weeks after the operation. Values are the mean ± standard deviation. *P < 0.05. MMP-3, matrix metalloproteinase-3; N.S, not significant.

**Figure 7:**
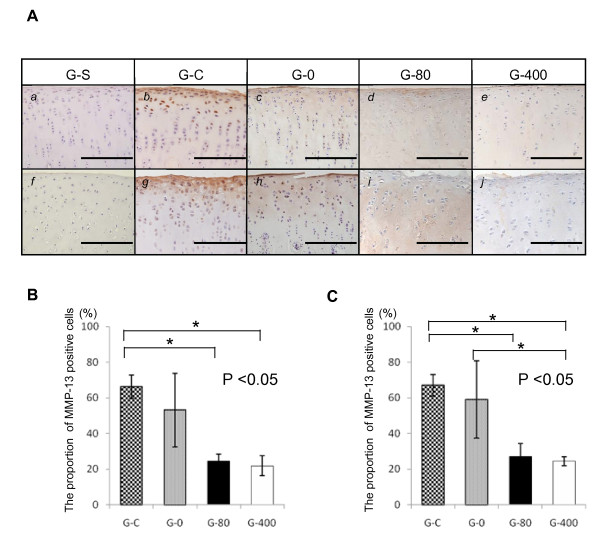
**Effect of ONO-8815Ly on the expression of MMP-13 in femoral condyles**. **(a) **Immunostaining for MMP-13 at two weeks after the operation. Sections of (*a *to *e*) medial and (*f *to *j*) lateral femoral condyles were prepared from samples of (*a *and *f*) G-S, (*b *and *g*) G-C, (*c *and *h*) G-0, (*d *and *i*) G-80, and (*e *and *j*) G-400. Magnification, 400×. Scale bar, 200 μm. Proportion of MMP-13-positive cells in **(b) **medial and **(c) **lateral femoral condyles at two weeks after the operation. Values are the mean ± standard deviation. *P < 0.05. MMP-13, matrix metalloproteinase-13; N.S, not significant.

MMP-3 and MMP-13 are major proteases degrading the ECM. The expression of these enzymes was analyzed by immunohistochemistry using samples prepared at two weeks after the operation. For MMP-3, there were no significant differences in staining intensity or number of positive cells between any of the groups (Figure 6). For MMP-13, however, significant differences were observed (Figure 7). The staining of MMP-13 was much stronger in G-C and G-0 (Figure 7a, b and 7c) than in G-S, G-80, or G-400 (Figure 7a, a, d, and 7e). The proportion of MMP-13-positive cells was significantly lower in sections of G-80 and G-400 than in sections of G-C or G-0 (Figure 7b). Similar results were obtained for the intensity (Figure 7a, f, i, and 7j) and the ratio of MMP-13-positive cells (Figure 7c) in the analyses of lateral condyles.

### EP2-selective agonist inhibits IL-1β-induced *MMP-13 *mRNA expression

**Figure 8:**
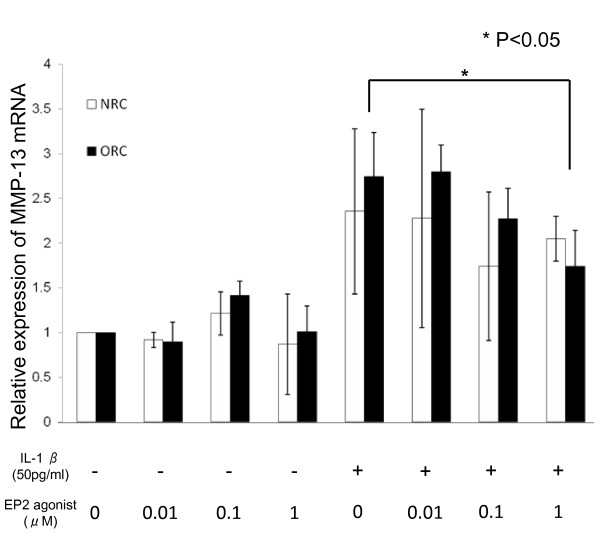
**Reduction of IL-1β-induced *MMP-13 *mRNA expression by an EP2 agonist on rabbit normal or OA chondrocyte primary culture cells *in vitro***. Expression levels of *MMP-13 *mRNA in NRC and ORC at six hours after treatment with IL-1β (50 pg/ml), ONO-AE-259-01, selective EP2 agonist (each at a concentration of 0, 0.01, 0.1, and 1 μM), or a combination of IL-1β and ONO-AE-259-01. GAPDH was used as internal control. Using the ratio obtained for the untreated sample as a standard (1.0), the relative ratio of the treated samples was presented as the relative expression levels of the *MMP-13 *gene. Values are the mean ± standard deviation. NRC (n = 4) and ORC (n = 5). *P < 0.05. EP2, prostaglandin E2 receptor type 2; IL-1β, interleukin-1β; MMP-13, matrix metalloproteinase-13; NRC, non-treated rabbit chondroctes; ORC, ACLMT-operated rabbit chondrocytes.

To confirm the effect of EP2 agonist on MMP-13 expression, the expression of the *MMP-13 *gene by primary cultured chondrocytes was evaluated by quantitative real-time PCR (Figure 8). The expression levels of *MMP-13 *were similar in NRC and ORC cells under basal culture conditions. Similarly, EP2 agonist treatment showed no significant effects on *MMP-13 *levels on either cells. When NRC and ORC cells were treated with IL-1β (50 pg/ml), the expression levels of *MMP-13 *mRNA were significantly increased in both cells. IL-1β-induced expression of MMP-13 mRNA in ORC cells was reduced by co-treatment with the EP2 agonist in a dose-dependent manner, and the maximum reduction was 37% at 1 μM of EP2 agonist. In the case of NRC cells, the maximum reduction (27%) was observed at the concentration of 0.1 μM.

## Discussion

The effect of PGE2 on the progression of OA is still a matter of debate. In some reports, PGE2 was shown to destroy articular cartilage by degrading cartilage ECM [12,13]. It has also been reported to down-regulate the production of IL-6 by IL-1α and IL-1β via EP2/EP4 receptors [25,26]. PGE2 at very low concentrations inhibits the production of IL-1β, TNF-α, and MMP-13 in the articular cartilages of OA patients [27]. In the current study, the production of MMP-13 was decreased by an EP2 agonist (Figures 7 and 8), which is consistent with the *in vitro *data described in a recent report [28]. Continuous administration of non-steroidal anti-inflammatory drugs to patients with OA exacerbates OA [29,30]. These contradictory results may be due to the differences in the experimental dose of PGE2 agonist used, or due to the pleiotropic effects of PGE2 through different types of receptors (EP1 to EP4). Therefore, analyses should be conducted with agonists specific for each type of receptor. IL-1β-induced expression of *MMP-13 *mRNA was reduced by EP2 signaling both in NRC and ORC cells *in vitro *(Figure 8). Moreover, IL-1β-induced expression of *MMP-13 *mRNA was reduced in ORC cells, but not in NRC cells, in a dose-dependent manner, that is, *MMP-13 *expression was higher in the presence of 1 μM of ONO-AE-259-01 than in the presence of 0.1 μM of ONO-AE-259-01 (Figure 8). An EP2 agonist acts as an anti-inflammatory drug at low doses, but if the concentration exceeds 1 μM, the anti-inflammatory effect may become weak (Figure 8). In fact, some authors have reported that excess EP2 agonists may act rather as inflammatory-inductive drugs.

Previously, we showed that EP2 signaling enhances the growth of chondrocytes [18,19] and promotes the regeneration of articular cartilage in rabbits with cartilage defects by an EP2-selective agonist [19]. However, in the current study, EP2 signaling failed to promote chondrocyte proliferation (Figure 5). The differences may result from differences in the animal models. In the previous study, the effect of EP2 signaling on articular cartilage was evaluated using the chondral and osteochondral defect models. In that model, cartilage defects are present before initiation of the treatment with an EP2 agonist. Thus, EP2 signaling may promote cartilage regeneration by inducing proliferation of cartilage chondrocytes and, consequently, contributing to ECM reconstruction. On the other hand, in the present study, the articular chondrocytes appeared normal immediately after the ACLMT operation, and EP2 signaling reduced cartilage degeneration caused by traumatic instability of the knee joint. These differences in models might be the cause of difference in the results.

In the present study, the abnormal stress on cartilage tissues induced by joint instability was the main cause of degeneration. The degeneration was more remarkable in the lateral (Figures 2d and 3d) than in the medial components (Figures 2c and 3c), wherein partial meniscectomy was performed. We have no clear explanation for this result. A study has shown that the lateral components of the rabbit knees were more susceptible to degeneration than the medial components in the ACLMT model [31]. The rabbit knee joints are physiologically in the valgus position, causing excess load on the lateral side, which might explain the susceptibility.

The grade of degeneration at 12 weeks was less prominent than we expected (Figure 4). In this injury model, cartilage degeneration will be induced by abnormal stress due to joint instability. Such abnormal stress takes place during weight-bearing movements of the knee joints. Therefore, to enhance such stress, Park et al. forced the rabbits to move in a confined space (5 m × 5 m) for one hour twice a day, from three days after ACLMT onward [22], which increased the Mankin's score up to 12 points at eight weeks after the operation. Restriction in a small cage in the knee-flexed position, as in our study, may minimize such stresses. In addition, both knees were operated on, which may further decrease the activities of the rabbits. These may cause almost no progression of the disease after two weeks.

Generally, cartilage degeneration in OA is due to the induction of MMP expression. MMP-13 is a product of chondrocytes that reside in cartilage and has a stronger effect that MMP-1 on type II collagen [32]. Some insisted that PGE2 exerts direct inhibitory effects on the expression of MMP-1 [33,34] and MMP-13 [28,33,34] in arthritic chondrocytes, and Sato et al. demonstrated that EP2 signaling was responsible for the down-regulation of MMP-13 *in vitro*, although they used a different agonist [28]. Taken together, EP2 signaling regulates MMP-13 production. In agreement, we showed that production of MMP-13 in articular chondrocytes was reduced when treated with an EP2 agonist *in vivo *(Figure 7) and *in vitro *(Figure 8). Controversially others studies show that PGE2 plays a crucial role in the induction of MMP-13 and MMP-3 in chondrocytes in response to IL-1β in microsomal prostaglandin E synthase-deficient mice [35] or that of PGE2 inhibits chondrocyte maturation [36]. In the current study model, EP2 signaling was shown to inhibit the expression of *MMP-13 *mRNA, suggesting that EP2 signaling protects the articular cartilage from degeneration.

MMP-3 is a protease expressed in OA specimens at an early stage [37,38]. MMP-3 cleaves a variety of ECM components such as proteoglycans, collagens, and procollagens [39]. In the current study, ONO-8815Ly had no effect on the production of MMP-3 (Figure 6). Although there is still much to be done, the current study suggested that an EP2 agonist may exert a protective effect on articular cartilage by inhibiting MMP-13.

It is important to clarify whether an EP2 agonist caused inflammation either systemically or locally. PGs are pro-inflammatory lipid mediators whose levels increase in the synovial membrane and synovial fluid of patients with OA. We previously reported that intra-articular administration of an EP2 agonist did not affect the mRNA expression of the *MMP-3*, *TIMP-3*, and *IL-1β *genes in the synovium, or the amounts of TNF-α and C-reactive protein (CRP) in joint fluids. As in our previous study, we found no severe inflammatory changes in the synovium, and no change in the levels of CRP (data not shown), suggesting that this EP2 agonist caused no inflammation either systemically or locally.

The effect of an EP2 agonist did not last long (Figure 4), yet this may be rectified by developing a suitable drug-delivery system. Continuous administration of an EP2 agonist using such a newly developed system could provide a novel therapeutic modality to treat OA.

## Conclusions

Stimulation of PGE2 via EP2 prevents degeneration of the articular cartilage during the early stages. The current study suggests that EP2 agonists may exert a protective effect on articular cartilage by inhibiting MMP-13. With a long-term delivery system, the EP2 agonist could be a new therapeutic tool for OA.

## Abbreviations

ACLMT: anterior cruciate ligament and menisectomy transaction; COX: cyclo-oxygenase; CRP: C-reactive protein; DMEM: Dulbecco's modified Eagle's medium; ECM: extracellular matrix; EP2: prostaglandin E2 receptor type 2; FBS: fetal bovine serum; GAPDH: glyceraldehyde 3-phosphate dehydrogenase; H&E: hematoxylin & eosin; IGF: insulin-like growth factor; IL: interleukin; MMP: matrix metalloproteinase; OA: osteoarthritis; PBS: phosphate-buffered saline; PCNA: proliferating cell nuclear antigen; PG: prostaglandin; PLGA: polylactic-co-glycolic acid; SD: standard deviation; TNF: tumor necrosis factor.

## Authors' contributions

HM performed animal experiments, carried out analysis and interpretation of the data, and drafted the manuscript. TA conceived this study, designed the study, carried out analysis and interpretation of the data, and drafted the manuscript. MF and JY performed animal experiments and carried out analysis of the data. KI performed animal experiments. TM was the chief investigator in the development of materials, and conceived this study. TK designed and performed animal experiments. SF performed animal experiments and obtained samples from animals. HS was responsible for providing materials. NA was responsible for the development of drug delivery system. TO carried out administrative and financial support and helped to draft the manuscript. TN carried out administrative and financial support and helped to draft the manuscript. JT conceived this study, provided financial support, designed experiments, interpreted the data, and drafted the manuscript. All authors have read and approaved the manuscript for publication.

## Competing interests

Takayuki Maruyama, Toshiya Kanaji, Shinsei Fujimura, Hikaru Sugihara, and Akio Nishiura are employees of Ono Pharmaceutical Co. Ltd. All other authors have no conflicts of interest.
